# Optimization of α-Fe_2_O_3_ Nanopillars Diameters for Photoelectrochemical Enhancement of α-Fe_2_O_3_-TiO_2_ Heterojunction

**DOI:** 10.3390/nano11082019

**Published:** 2021-08-07

**Authors:** Herme G. Baldovi

**Affiliations:** Department of Chemical Engineering and Biotechnology, University of Cambridge, Philippa Fawcett Drive, Cambridge CB3 0AS, UK; hergarba@cam.upv.es

**Keywords:** hematite, nanopillars diameter, electron beam evaporation, water splitting, TiO_2_ support, hydrogen, heterojunctions

## Abstract

Global warming is pushing the world to seek to green energy sources and hydrogen is a good candidate to substitute fossil fuels in the short term. In future, it is expected that production of hydrogen will be carried out through photo-electrocatalysis. In this way, suitable electrodes that acts as photoanode absorbing the incident light are needed to catalyse water splitting reaction. Hematite (α-Fe_2_O_3_) is one of the most attractive semiconductors for this purpose since it is a low-cost material and it has a suitable band gap of 2.1 eV, which allows the absorption of the visible region. Although, hematite has drawbacks such as low carrier mobility and short holes diffusion lengths, that here it has been tried to overcome by nanoengineering the material, and by using a semiconductor as a scaffold that enhances charge carrier separation processes in the electrode. In this work, we fabricate ultrathin quasi transparent electrodes composed by highly ordered and self-standing hematite nanopillars of a few tens of nanometers length on FTO and TiO_2_ supports. Photoanodes were fabricated utilizing electron beam evaporation technique and anodized aluminum oxide templates with well-defined pores diameters. Thus, the activity of the compact layer hematite photoanode is compared with the photoanodes fabricated with nanopillars of controllable diameters (i.e., 90, 260 and 400 nm) to study their influence on charge separation processes. Results indicated that optimal α-Fe_2_O_3_ photoanodes performance are obtained when nanopillars reach hundreds of nanometers in diameter, achieving for photoanodes with 400 nm nanopillars onto TiO_2_ supports the highest photocurrent density values.

## 1. Introduction

Growth in population together with global economy is related to an increase in global energy demand [1,2,3]. Currently, more than 80% of the primary energy is obtained by fossil fuels (coal, natural gas and oil. Despite this, fossil fuels are limited sources that will not be able to supply the future energy demand. Furthermore, their processing, transport and use contribute to the global climate change and environmental damage [2,4,5]. In this way, renewable energy sources are becoming more popular since they are unlimited and considered as clean energy sources with no toxic emissions [3,6].

**Figure 1:**
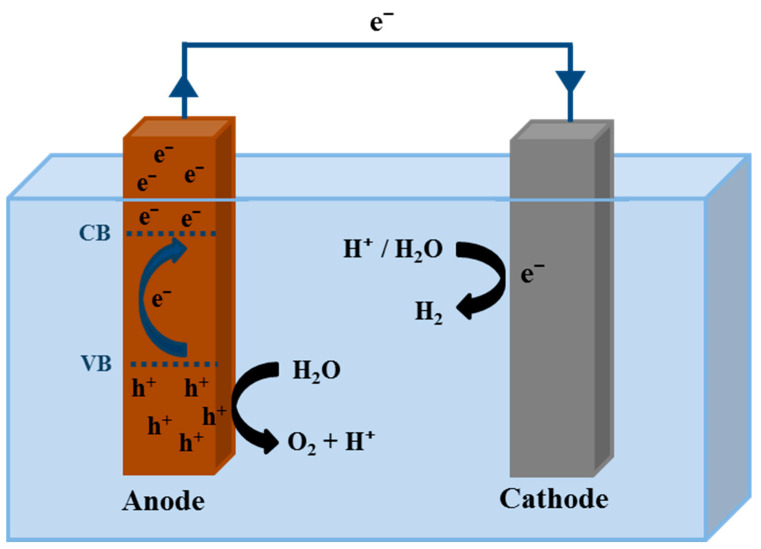
Schematic representation of a PEC cell.

Hydrogen produced from renewable energy sources is one of the alternatives to solve the environmental problem and the energy crisis. Hence, a technology towards the production of hydrogen from renewable and sustainable energy resources is one of the scientific challenges today [3,6,7]. Different methods are being investigated to produce O_2_, among them, photoelectrochemical (PEC) water splitting is one of the most interesting technologies. It only needs solar energy and water as primary sources, and no toxic emissions or sub-products are released to the environment [3,4]. In Figure 1 PEC cell is depicted.

A PEC cell consists of an anode and a cathode that are immersed in a conductive electrolyte and also connected by an external circuit [6,8,9]. One or both electrodes need to be photoactive materials to photogenerate electron-hole pairs when illuminated [10]. As a basic fundamental of the PEC cell, when the photoanode is illuminated by solar light, electron-hole pairs are photogenerated, and a charge separation is produced. Then, the holes go to the anode/electrolyte interface to oxidize the water to *O*_2_ according to Equation (1) [11].

(1) $$H_{2}O+2h^{+}\to 2H^{+}+\frac{1}{2}O_{2}$$

whereas the electrons move from the valence energy band (VB) to the conduction energy band (CB) of the anode. Therefore, they can go through the external circuit arriving to the cathode, and in the cathode/electrolyte interface they reduce the water to produce *H*_2_ according to Equation (2) [11].

(2) $$2H^{+}\;+2e^{-}\to H_{2}$$

The most important components of a PEC cell are the anode and cathode. However, the Oxygen Evolution Reaction (OER) at the anode is slower than the hydrogen production at the cathode, limiting the PEC process [5]. Then, the study and optimization of the photoanode is a key factor in the photon-to-hydrogen conversion efficiency in the PEC water splitting process [12]. Since Fujishima and Honda demonstrated that the water electrolysis was possible by using a semiconductor such as TiO_2_ as photocatalyst [13], a lot of works with different metal oxide semiconductors have been developed [14,15,16,17]. Actually, most of these studies are focused on semiconductors that are only active in UV light since it has considerably more energy per photon than visible light [18]. Nevertheless, considering that UV light only represents 4% of the solar spectrum while visible light reaches the 53% approximately, it is more interesting to develop materials that also are capable to harvest visible light. In this way, even less efficient photoanodes responding to the visible light can reach higher results than the ones that only respond to the UV light [19,20].

α-Fe_2_O_3_ is the most stable iron oxide compound material and is widely used in photoelectrodes, gas sensing, catalysts, magnetic recording, and medical fields. Hematite (α- Fe_2_O_3_) is one of the most attractive materials for obtaining hydrogen from solar energy due to its properties [21,22]. Hematite has a band gap of 2.1 eV, which allows absorbing visible light up to 600 nm in the solar spectrum, and it has good chemical stability in aqueous alkaline environments. Moreover, it is a no toxic, environmentally friendly, abundant and low-cost material [9,21,23,24,25]. All these properties make hematite suitable for act as photocatalyst in PEC water splitting. Nonetheless, its low carrier mobility (0.01–0.1 cm^2^·V^−1^·s^−1^) and short hole diffusion length (2–4 nm) leads to fast recombination (10^−12^ s), reducing considerably its efficiency [23,26,27,28]. To address these limitations and improve solar conversion efficiency, enormous efforts have been focused on the synthesis of hematite nanostructured films, modification of electronic structure via elemental doping, passivation or coupling with electrocatalyst such as IrO_2_ [29,30]. Other strategy to overcome these issues is nanostructuring because nanostructures provide specific routes for redirecting electrons and holes, thereby reducing the recombination rate [31,32]. Also, nanostructures have higher surface areas than a compact layer of hematite, so the anode/electrolyte interface is higher improving the PEC process efficiency [27,33,34]. Recently other research groups have developed similar nanostructured hematite nanopillar arrays photoanodes and studied that the formation of heterojunctions leads to significantly improve the transfer of charge transfer. For instance, Sha-Sha Yi et al. [35] claim that Fe_2_o_3_ passivation with In_2_O_3_ leads to a decrease of charge recombination facilitating efficient charge separation. Other researchers such as Bo Lei et al. [36] have significantly improve photo-electrocatalytic efficiency by the decorating hematite nanopillars surface with magnetite (Fe_3_O_4_) with an excellent conductivity and suitable energy bands positions, these heterojunctions lead to the improve of interfacial charge-transfer efficiency. Hence, hematite nanostructures are promising materials for being used as photocatalysts in PEC water splitting for producing clean hydrogen.

In this work, we have prepared nanostructured photoanodes made of highly ordered hematite nanopillars array with controlled diameters with the aim: firstly, study light harvesting capacity due to nanostructuration and to nanopillar diameter; secondly, understanding of dependence of the efficiencies of charge separation and charge mobility processes in function of nanopillar diameter; and finally, study the effect of Fe_2_O_3_-TiO_2_ heterojunction into the photoanode performance. In our case TiO_2_ layer is underneath of the hematite nanopillar that are made using electron beam (e-beam) evaporation and anodic aluminum oxide (AAO) templates. E-beam evaporation technique was selected because it is a promising method to synthesize metal oxide nanostructures due to its simplicity and scalability [37]. The iron nanopillars were synthesized on two different substrates: fluorine doped tin oxide (FTO) transparent conductive glass and titanium foil. Titanium substrates were chosen since having a semiconductor such as TiO_2_ (a thin superficial layer of Ti will passivate to TiO_2_ after the annealing of the electrodes) as supports can improve charge carrier separation processes in the iron oxide electrode [27]. The aim of the study is to use electron beam evaporation together with AAO templates to form highly ordered α-Fe_2_O_3_ nanopillars and study the influence of the different controllable diameters on the properties of the electrodes. In this way, the nanostructures were examined morphologically by means of Scanning Electron Microscopy (SEM), and structurally by X-ray Photoelectron Spectroscopy (XPS), X-ray Diffraction (XRD) and diffuse reflectance UV-Visible (UV-Vis) spectroscopy. Finally, the photoelectrochemical properties of the electrodes were studied by the current/photocurrent density versus applied potential curves and chopped-light chronoamperometry plots. Also, Inductively Coupled Plasma Optical Emission Spectroscopy (ICP-OES) was performed to measure the amount of iron oxide present in the studied electrodes. Thus, a complete characterization of the synthesized hematite electrodes and a comparison between the two studied supports are shown in this work.

## 2. Experimental

### 2.1. Preparation of the Substrates with the AAO Templates

For the preparation of the different conductive substrates, FTO glass (Sigma-Aldrich, Merck KGaA, San Luis, MO, USA) with a surface resistivity of ~7 Ω/sq and titanium foils (Alfa Aesar, Haverhill, MA, USA) of 0.127 mm thick with a purity of 99.99% metal basics were cut into 2 cm × 1 cm pieces. Afterwards, in order to remove any kind of contamination, the substrates were sonicated in different baths for 15 min each one: ultrapure water (USFELGA, 18.2 MΩ), isopropanol (VWR, 99.9%) and acetone (VWR, 100%), respectively. Then, they were dried with nitrogen stream and thereby prepared for the sticking of the AAO templates.

The commercial ultrathin AAO templates (Topmembranes Technology, Shenzhen, China) had a layer of Poly (methyl methacrylate) (PMMA) to facilitate the template sticking process on the substrates. Thus, the AAO templates together with the substrates were put in different acetone baths (in order to dissolve the PMMA) until the templates were completely stuck on the substrates with any PMMA left. Templates with different diameters, i.e., 90, 260 and 400 nm, were used to study their influence on the Fe_2_O_3_ nanopillars formation.

### 2.2. Preparation of α-Fe_2_O_3_ Electrodes

Electron beam evaporator (Kurt J. Lesker) with power supply (M.A.P.S. EBPS-A) was used for the deposition of metallic iron on the different substrates with the AAO templates to form the iron nanopillars with specific diameters. The area of the electrode was fixed to 5 mm × 5 mm for all the samples by covering the uninterested parts with polyamide tape. Iron pellets 99.99% pure (Kurt J. Lesker) were used as metallic source for the electron beam evaporation. Prior to evaporation, the chamber was evacuated to a pressure of 1.5 × 10^−5^ Torr and maintained to this value during iron evaporation. For all the samples, were evaporated 40 nm of pure metallic iron with a deposition rate of 0.15 Å/s, and the sample holder was rotating during the evaporation at 0.1 rpm. To carry out the e-beam evaporation, a hot tungsten filament was used to generate electrons which, after acceleration with 1 ampere and 10 kV, can provide electrons with 10 kW of energy capable of melting high melting point metals. In the case of our electron beam evaporator, the deposition rate was controlled by a microbalance that regulates the current at the anode (where the crucible with the Fe source is located) and the electron acceleration voltage which can vary from 3 to 40 kV. Thus, depending on the accelerating voltage and current, the impact of the electrons on the source, and therefore the amount of metal evaporated and deposited, can be controlled. Also, iron deposition on FTO and Ti substrates without templates were done with the aim of synthesizing a compact layer as control and comparing its photoelectrochemical behaviour with those of the nanostructures.

**Figure 2:**
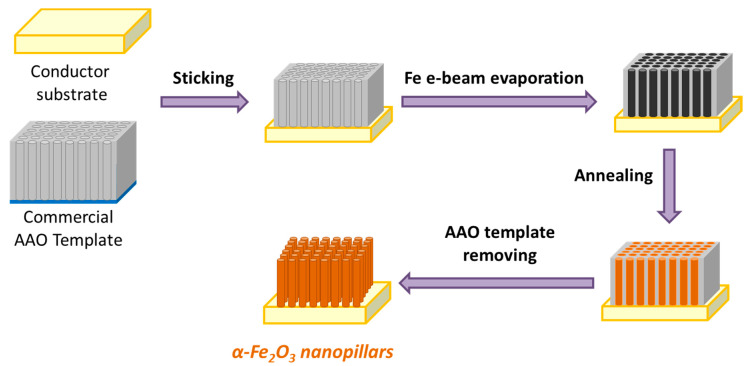
Schematic representation of the experimental procedure to fabricate self-standing α-Fe_2_O_3_ nanopillars with controllable diameters, by using e-beam evaporation technique with AAO templates.

After the electron beam evaporation, samples were annealed in a quartz tube furnace (Carbolite, Type MTF12/50/250, Sheffield, Reino Unido) with a temperature controller (Eurotherm 91e, Worthing, Reino Unido) for 1 h at 500 °C with a 2.5 L·min^−1^ air flow. Once the samples were cooled down, part of the templates were separated from the substrates due to the temperature difference. However, the samples were immersed in 1 M NaOH (Sigma Aldrich, Merck 100.5%) solution for 2 h at room temperature, to completely dissolve the AAO templates in order to have self-standing nanopillars. The experimental procedure carried out to synthesize the α-Fe_2_O_3_ nanopillars is depicted in Figure 2.

### 2.3. Morphological and Structural Characterization

Scanning electron microscopy. Samples were morphologically examined by means of a Scanning Electron Microscope (TESCAN, Mira3) with a high voltage of 5.0 kV and an In-Beam SE detector.

X-ray photoelectron spectroscopy. The surface chemistry of the iron oxide layer were analyzed by XPS using a spectrometer (SPECS) with a 150-MCD9 detector using MgKα (*hν* = 1253.6 eV) X-ray radiation at an energy of 50 W.

X-ray diffraction analysis. Analysis of iron oxide sample was carried out with a X-ray diffractometer using CuKα (λ = 1.54 Å) radiation, operating in reflection, theta-theta mode with a 2D strip detector.

Diffuse reflectance UV-Vis spectroscopy. Diffuse reflectance ultraviolet and visible spectres of iron oxide layer were recorded with a spectrophotometer (Agilent Cary 300) coupled to a Diffuse Reflectance Accy Internal, 70 mm Cary integrating sphere.

### 2.4. Photoelectrochemical Characterization

Photoelectrochemical characterization was performed in a home-made photoelectrochemistry (PEC) cell using a three electrodes configuration with the iron oxide samples as the working electrodes, a platinum wire as the counter electrode and an Ag/AgCl (3 M KCl) electrode as the reference. The electrolyte for all the measurements was 1 M NaOH and the PEC cell was irradiated by a 500 W Xe lamp (Oriel instrumentsNewport, Irvine, CA, USA) for the illumination conditions. On the one hand, current/photocurrent density values were measured for each sample from −0.4 to 0.6 V (vs. Ag/AgCl) with a scan rate of 2 mV/s. The samples were tested under dark conditions, with a visible filter (Newport) that cut the light below 420 nm and with a solar AM 1.5 G filter (Oriel instruments) (55 mW·cm^−2^). On the other hand, chopped-light chronoamperometry measurements were acquired at 0.5 V (vs. Ag/AgCl) under dark, visible (λ > 420 nm) and solar AM 1.5 G (55 mW·cm^−2^) conditions.

Finally, the content of iron oxide in the samples with titanium supports was measured by the inductively-coupled plasma optical emission spectroscopy (Perkin Elmer Optima 2100 DV, Waltham, MA, USA). Samples were digested in aqua regia (3:1 concentrated HCl:HNO_3_) and then, the solutions were diluted with ultrapure water previous to the ICP-OES analysis. The instrument was calibrated beforehand by a suitable ICP standard solution.

## 3. Results and Discussion

### 3.1. Morphological and Structural Characterization

Electrodeposition is commonly utilized for the formation of iron oxides films in literature, however, with this method is difficult to prepare films of a few nanometers thick and due to the appearance, on the electrode, of hot spots with less electric resistance will make difficult to have a homogeneous layer in thickness. Since iron has a high boiling point of about 2862 °C is not suitable to make iron films with a current thermal evaporator. In this sense, to perform high quality photoanodes it was utilized electron-beam physical vapor deposition (EBPVD) that evaporates iron by bombarding the iron source with an electron beam as the heating source under ultrahigh vacuum. EBPVD technique is an attractive option for the fabrication of nanostructures made of metals with high boiling temperatures since it is a simple technique and easily scalable.

**Figure 3:**
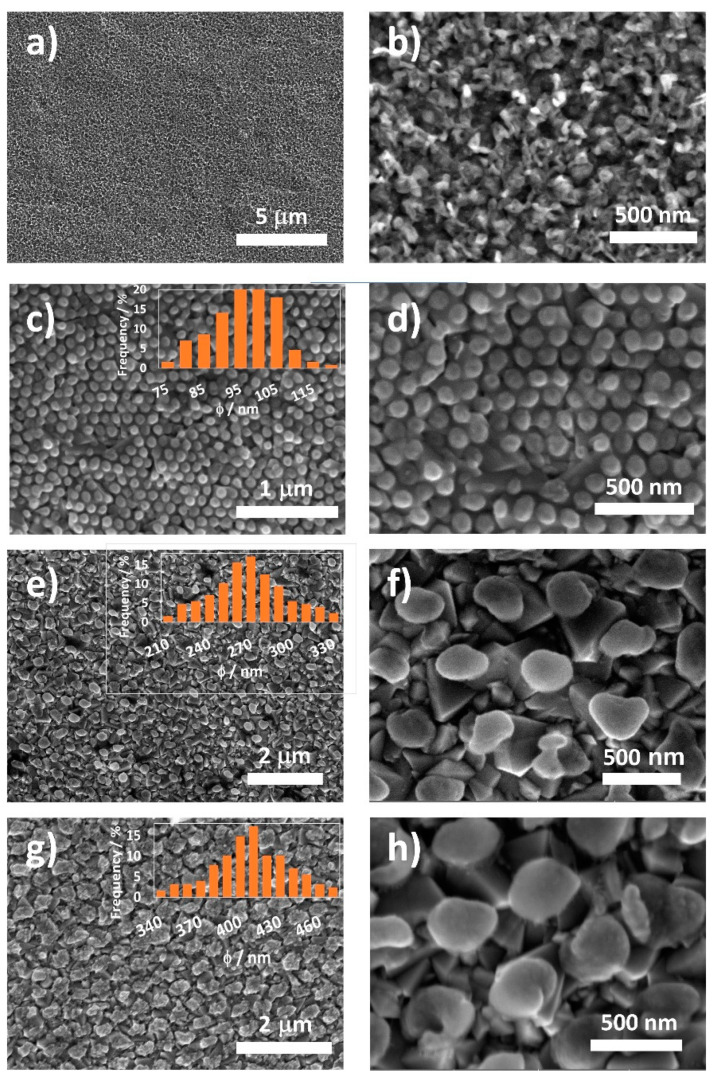
SEM top images of an iron oxide compact layer (**a**,**b**) synthesized by e-beam evaporation directly on a FTO substrate, and iron oxide nanopillars synthesized by e-beam evaporation using AAO templates with different diameters: φ90 nm (**c**,**d**), φ260 nm (**e**,**f**) and φ400 nm (**g**), (**h**) on FTO substrates.

Iron oxide nanopillars were synthesized on FTO substrates in order to check the viability of the e-beam evaporation technique combined with the use of pre-made AAO templates to form highly ordered and self-standing α-Fe_2_O_3_ nanopillars. Figure 3 shows SEM images at several magnifications of the different nanostructures synthesized by e-beam evaporation on FTO substrates. On the one hand, in Figure 3a,b it is seen that after e-beam evaporation of the iron directly on the FTO substrate (without AAO template) a compact layer (CL) is formed. This CL is non-homogeneous in surface because the iron oxide growth is not completely regular and because of rugosity of the FTO, then little imperfections are present on the surface of the CL. These imperfections could act as recombination centers (carrier trap centers) favoring the recombination of the generated electron-hole pairs, which can be detrimental for the efficiency of the photoanodes in photoelectrochemical applications.

On the other hand, when AAO templates with known diameter values are used in combination with e-beam evaporation, highly ordered and self-standing nanopillars with specific diameters were formed. Some authors synthesized nanostructures by e-beam evaporation varying the incident angle [38], and obtained nanostructures with particular morphologies. However, in this work, we present the novelty of synthesizing self-standing iron oxide nanopillars with specific diameters by using e-beam evaporation in combination with AAO templates. Therefore, since the AAO templates were filled during the e-beam evaporation, the diameters of the nanopillars were perfectly controllable.

During this study were prepared nanopillars with diameters from 90 to 400 nm. Figure 3c,d shows nanopillars formed with AAO templates of φ90 nm, and an average diameter of φ95 ± 12 nm was obtained for the nanopillars. These nanopillars are completely homogeneous on the FTO surface, which is beneficial for the photoelectrochemical applications. When an AAO template of 260 nm in diameter was used, nanopillars with φ265 ± 33 nm were obtained (Figure 3e,f). Finally, when AAO templates 400 nm were chosen, nanopillars with an average diameter of φ409 ± 51 nm were formed (Figure 3g,h). In the three cases, FTO crystals can be appreciated underneath the nanopillars because of the magnification of the SEM images (see Figure 3d,f,h). These crystals can be more noticed in the samples with nanopillars having diameters of 260 and 400 nm, since the physical space between nanopillars are higher than those of the nanopillars with lower diameter (90 nm).

**Figure 4:**
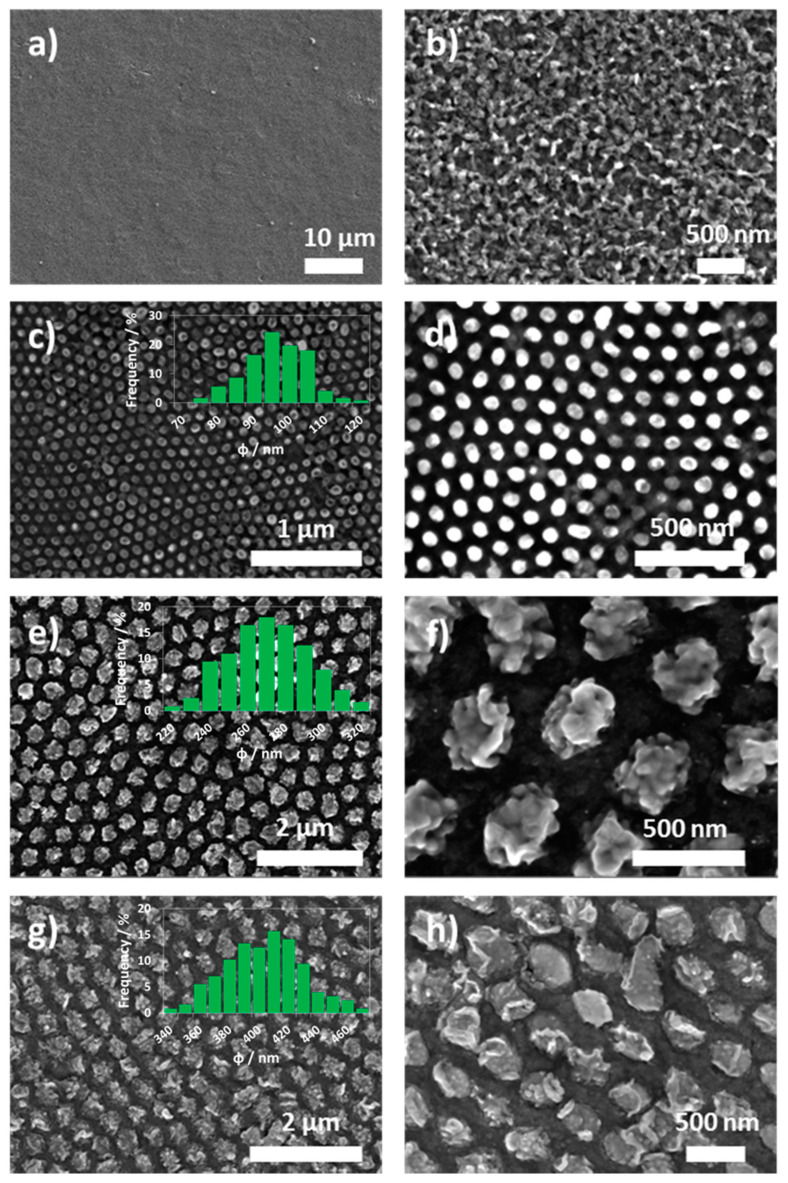
SEM top images of an iron oxide compact layer (**a**,**b**) synthesized by e-beam evaporation directly on a Ti foil, and iron oxide nanopillars synthesized by e-beam evaporation using AAO templates with different diameters: φ90 nm (**c**,**d**), φ260 nm (**e**,**f**) and φ400 nm (**g**,**h**) on Ti foils.

Regarding to the samples synthesized on Ti foils, Figure 4 shows the SEM top view images of the different nanostructures. Figure 4a,b indicates that a compact layer was formed when no template was used during e-beam evaporation of iron. The compact layer was also non-homogenous in the surface as occurred when using the FTO substrates probably due to its high rugosity. Nevertheless, when AAO templates were used during the e-beam evaporation, nanopillars with different diameters were obtained. These nanopillars had diameters of 92 ± 12, 271 ± 34 and 405 ± 51 nm when using AAO templates that were 90, 260 and 400 nm in diameter, respectively. These diameter values indicated the high controllability of the nanopillars obtained by e-beam evaporation owing to the AAO templates. In contrast to FTO substrate, while nanopillars grow in diameter same rugosity, as seen in the compact layer, begin to appear on the top part of the pillar.

Furthermore, it must be said that the space between the nanopillars corresponds to the titanium foil used as substrate. Therefore, since the annealing temperature for the formation of crystalline iron oxide nanopillars was 500 °C, titanium foil gets passivated forming titanium oxide. According to Hou et al. [39] the formed TiO_2_ film at this annealing conditions should correspond to anatase crystalline phase [40]. In this case, scanning electron images of the α-Fe_2_O_3_ nanopillars show that dispersed in an ordered way and are sitting on top of the TiO_2_ film generating an effective heterojunction that will enhance the photoelectrochemical response of the electrodes [41].

As a first structural characterization, XPS analysis was performed before and after the annealing of the samples. The aim was to check that all metallic iron (Fe^0^) which was deposited on the substrates was oxidized after the heating treatment. Figure S1 (supporting information section) shows the obtained spectra with Fe^0^, 2p^1/2^, 2p^3/2^ and satellite peaks [42,43,44]. The peak associated to Fe^0^ (at 706.5 eV) disappeared after the annealing at 500 °C for 1 h, indicating that all metallic iron was oxidized as expected.

In order to see the crystalline structure of the resultant iron oxide layers of the electrodes, XRD analysis was carried out, shown in Figure S2 (supporting information section). The XRD pattern exhibited peaks at 2θ range of 24.55°, 33.55°, 36.01°, 41.26°, 49.85°, 54.44°, 57.92°, 62.74° and 64.34°, which are associated to (012), (104), (110), (113), (024), (116), (018), (214) and (300), respectively. All these peaks correspond to lattice planes of hematite (α-Fe_2_O_3_) [45,46,47]. Furthermore, the narrow sharp peaks of hematite and the absence of diffraction peaks of other phases in the XRD pattern indicated the high crystallinity and purity of the obtained hematite. Thus, the iron oxide of the photoanodes was in its α-Fe_2_O_3_ crystalline form.

The diffuse reflectance UV-Vis spectrum of the samples is shown in Figure S3a) (supporting information section), illustrating the characteristic UV-Vis spectrum of hematite. From the spectra, both direct and indirect band gap energies were calculated using Tauc’s equation (Equation (3)) [9,32]:

(3) $$\left(\alpha \cdot h\cdot \nu \right)=A\cdot {\left(h\cdot \nu -E_{g}\right)}^{n}$$

where α is the absorption coefficient, *h*·*ν* is the photon energy (in eV), *A* is a proportional constant, *E_g_* is the band gap energy (in eV) and n is a constant that depends on the nature of the electron transition. For a direct allowed transition *n* = ½ and for an indirect allowed transition *n* = 2. Thus, (*α*·*h*·*ν*)^2^ vs. photon energy was plotted in order to estimate the direct band gap energy of hematite (see Figure S3b), whereas (*α*·*h*·*ν*)^1/2^ vs. photon energy was plotted to evaluate the direct band gap energy (shown in Figure S3c). From the plots, a direct *E_g_* of 2.2 eV and an indirect *E_g_* of 2.15 eV were estimated for the samples, which are consistent with reported values in literature for hematite [20,48,49].

### 3.2. Photoelectrochemical Characterisation

Photoelectrochemical performance of a-Fe_2_O_3_ photoanodes were tested under dark conditions and utilizing two regions of light: Visible (λ > 420) and simulated solar light. Thus, FTO substrates were chosen since this conductive crystal is transparent at wavelengths superior to middle wave ultraviolet (λ > 310 nm [50]). Hence, α-Fe_2_O_3_ is the only responsible of the photoanode behavior. In this way, we are able to compare photoanode photoresponse between compact layer and nanostructured anodes, optimal nanopillar diameter and their response to different regions of the light spectra.

**Figure 5:**
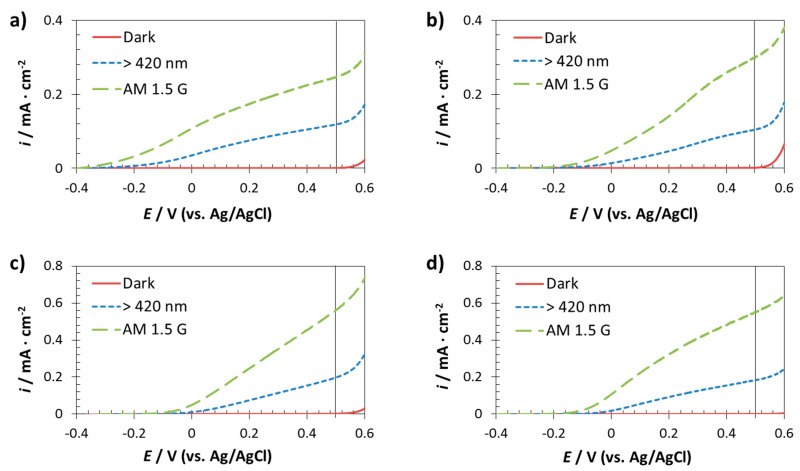
Current/photocurrent density versus applied potential curves of the photoanodes fabricated on FTO substrates: compact layer (**a**) and using different AAO templates: φ90 nm (**b**), φ260 nm (**c**) and φ400 nm (**d**). Dark, visible light (λ > 420 nm) and solar AM 1.5 G (55 mW·cm^−2^) illumination conditions were applied.

The prepared electrodes on FTO were photoelectrochemically characterized by current/photocurrent density versus applied potential curves. Samples were measured in a 1 M NaOH solution since the stability of iron oxide in alkaline media is excellent [25,31]. Figure 5 shows the different curves obtained for the electrodes on FTO substrates, measured under dark, visible filter and simulated solar light reaching the Earth surface.

It is noticed that under dark conditions there was no response for any of the samples. This was because illumination is needed in a photoelectrochemical process in order to generate electron-hole pairs, which are required for having a flow current inside the electrode [24]. In particular, the incident light must have photon energies larger than the band gap energy of the semiconductor [51], in this case the iron oxide electrodes, which have a band gap of 2.2 eV. Thus, the higher the incident energy, the higher the obtained photocurrent density is. For that reason, Figure 5 shows that all the electrodes showed higher photocurrent density values under solar AM 1.5 G illumination than under visible illumination.

Nevertheless, dark curves give information about the stability of the materials under different applied potentials. Hence, if the electrode does not show any response under dark conditions is because no detriment of the surface is taking place. Thus, Figure 5 indicates that the studied electrodes were stable when applying 0.5 V (vs. Ag/AgCl) (see vertical line at 0.5 V in the plots). Consequently, 0.5 V (vs. Ag/AgCl) was fixed as a potential value for the subsequent chopped-light chronoamperometry measurements, which give information about the viability of the iron oxide electrodes as photocatalysts in water splitting. Additionally, it was appreciated that on FTO, that more cathodic open circuit potentials were obtained for the nanostructured photoanodes implying that these photoanodes will improve water oxidation capacity compared to non-nanostructured photoanodes

**Figure 6:**
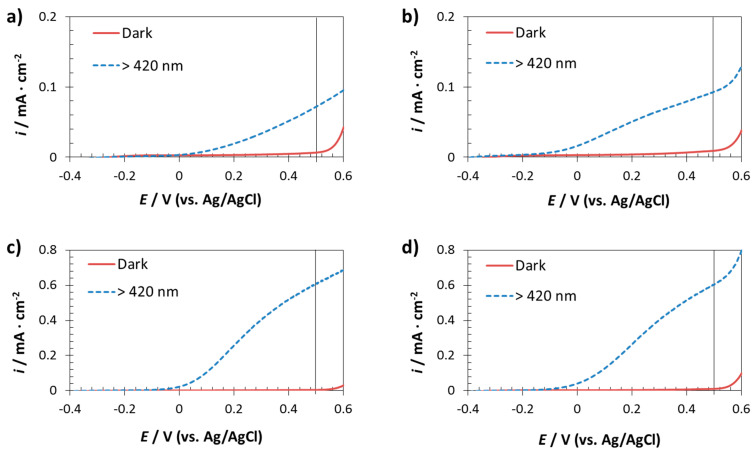
Current/photocurrent density versus applied potential curves of the photoanodes fabricated on Ti substrates: compact layer (**a**) and using different AAO templates: φ90 nm (**b**), φ260 nm (**c**) and φ400 nm (**d**). Dark and visible light (λ > 420 nm).

A TiO_2_ support was used in this study since it is a semiconductor with suitable photoelectrochemical behavior due to its wide band gap 3.2 eV for anatase [52,53] allowing to respond only under UV illumination. The aim was to evaluate performance of the Fe_2_O_3_ nanopillars to study how heterojunctions affects charge carrier separation among the nanostructured α-Fe_2_O_3_ samples due to the TiO_2_ supports underneath. Although these photoanodes were analysed under visible and simulated solar light, to study charge separation phenomena of α-Fe_2_O_3_, was paid more attention into the photoanodes analysed under visible light. Figure 6 illustrates the current/photocurrent density versus applied potential curves of the fabricated electrodes on titanium substrates under dark and visible light (λ > 420 nm). As Figure 6 shows, the photocurrent density values obtained for the nanopillars were higher than those reached for the compact layer. In the case of φ260 and φ400 nm nanopillars photoresponse at 0.5 V were six times higher than for compact layer photoanodes. Moreover, all samples made of nanopillars structure has more negative open circuit potentials than of compact layer. Stability iron oxide film was also confirmed submitting these samples to 1 h constant light irradiation at 0.5 V.

**Figure 7:**
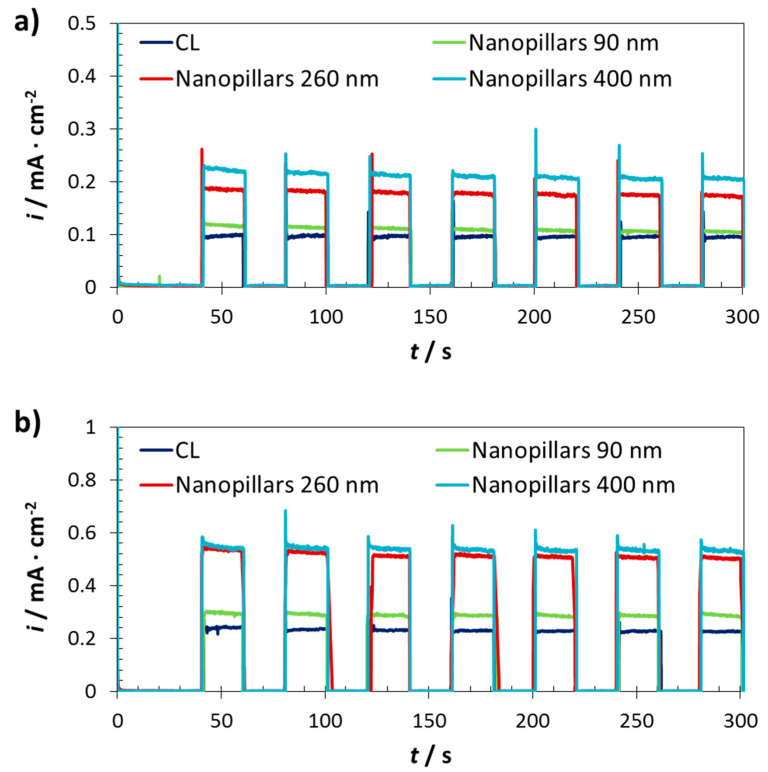
Chopped-light chronoamperometry measurements at 0.5 V (vs. Ag/AgCl) of the photoanodes fabricated on FTO substrates, under visible light (λ > 420 nm) (**a**) and under solar AM 1.5 G (55 mW·cm^−2^) illumination (**b**).

Chopped-light chronoamperometry measurements were carried out in 1 M NaOH at 0.5 V (vs. Ag/AgCl), according to the information obtained by the current/photocurrent density versus applied potential curves. Figure 7 shows the pulses obtained under visible (λ > 420 nm) and solar AM 1.5 G light conditions. Figure 7a shows the pulses obtained for all the samples when illuminating with visible light, in particular with irradiation wavelengths equal or higher than 420 nm. It is clearly seen that the iron oxide compact layer showed the lowest photocurrent density values (~0.08 mA·cm^−2^). By contrast, the iron oxide nanopillars achieved higher values due to the fact that this morphology enhances the charge carrier separation. On the one hand, nanopillars provide a direct longitudinal pathway to the electrons for arriving rapidly to the conductor substrate and then, to the cathode to reduce water. On the other hand, unlike compact layer, the specific diameters of the nanopillars allow the holes to easily move to the electrode/electrolyte interface to oxidise water. According to this, the recombination rate is considerably reduced in comparison to having a compact layer. Figure 7 indicates that the larger the diameter of the nanopillars, the higher the photocurrent density reached. Thus, the nanopillars having diameters of 400 nm obtained the best results achieving more than 0.2 mA·cm^−2^.

Regarding the pulses obtained for the samples when illuminating with solar AM 1.5 G light, shown in Figure 7b, the trend was the same, but all the reached photocurrent density values were higher than those achieved under visible light. This trend was as expected since visible light accounts 53% of the total solar energy [19]. Therefore, by using a solar filter, UV light is also contributing to the global photoelectrochemical process. The nanopillars φ400 nm reached the highest photocurrent density values (0.54 mA·cm^−2^), but these values were very similar to the ones achieved for the nanopillars φ260 nm (0.51 mA·cm^−2^).

When comparing to the literature, it is clear that the achieved photocurrent density values indicate an advance for the iron oxide nanopillars/nanorods in the photoelectrochemical field. Some authors worked with pristine iron oxide nanorods, achieving photocurrent density values of the order of 0.01 [54], 0.09 [29] or 0.49 [33] mA·cm^−2^, measured at similar conditions, i.e., 0.5 V (vs. Ag/AgCl) with solar AM 1.5 G illumination. However, the power used (100 mW·cm^−2^) was almost twice that used in this study (55 mW·cm^−2^). Furthermore, although the latter result is slightly lower than that obtained in this work for the nanopillars φ400 nm under solar AM 1.5 G (55 mW·cm^−2^) illumination, the key of the present study is that the nanopillars were 40 nm in length, which was a very short value for such a great photocurrent density value. This demonstrated that the pristine iron oxide nanopillars synthesized in this work achieved notable photoelectrochemical results.

**Figure 8:**
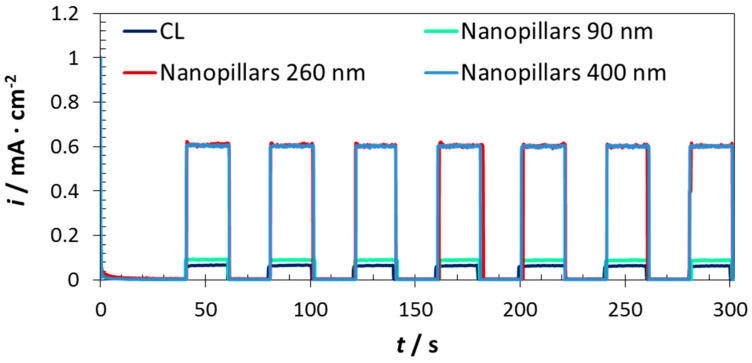
Chopped-light chronoamperometry measurements at 0.5 V (vs. Ag/AgCl) of the photoanodes fabricated on Ti substrates, under visible light (λ > 420 nm).

Chopped-light chronoamperometry measurements of the iron oxide nanopillars on TiO_2_ supports were shown in Figure 8. The trend is in agreement with that shown in Figure 7. On the one hand, the compact layer and nanopillars φ90 nm showed similar photoelectrochemical response (~0.09 mA·cm^−2^ in both cases). On the other hand, nanopillars φ260 and φ400 nm presented higher photocurrent density values, which were of the order of 0.6 mA·cm^−2^, but without considerable differences between both diameters.

In comparison with the results presented in Figure 7a, synthesizing the iron oxide nanopillars on TiO_2_ supports supposed an enhancement of almost three times with respect to doing it on FTO. Furthermore, the results presented in Figure 8 were even higher than those presented in Figure 7b. This means that the photocurrent density reached for the nanopillars φ260 and φ400 nm when illuminated with visible light are higher than those obtained for the nanopillars synthesized on FTO substrates when illuminated either by visible or solar simulated light.

**Scheme 1:**
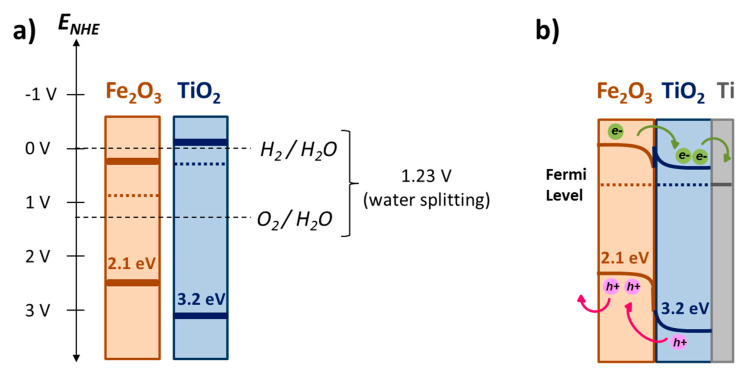
Energy bands positions of Fe_2_O_3_ and TiO_2_ semiconductors when isolated (**a**) and when they are connected (**b**).

This noticeable improvement is attributed to the dual effect of Fe_2_O_3_ and TiO_2_. When both semiconductors are connected, they reach the equilibrium and thereby the same Fermi level (see Scheme 1), changing the position of their energy bands [41,55,56]. In this way, when the electrode is illuminated with visible light, the photogenerated electrons on Fe_2_O_3_ can transfer from the conduction band (CB) of Fe_2_O_3_ to the CB of TiO_2_ (which is below the CB of the Fe_2_O_3_). At that moment, the electrons can easily go through the thin TiO_2_ layer to metal form titanium and then to the cathode in order to produce H_2_ (according to Equation (2)). Then, having a TiO_2_ supports captures the photogenerated electrons from the host α-Fe_2_O_3_, reducing then the electron-hole recombination on the electrode [57] and enhancing the photoelectrochemical behavior of the electrode. Figure S4 compares the photocurrent densities for all samples in FTO and titanium foil when are excited with visible light. Showing a clear evidence that heterojunctions lead to remarkable improve in the photoanode performance. This indicates that charge separation and charge transfer was enhanced due to the formation of TiO_2_-Fe_2_O_3_ heterojunction. This fact is also supported when incident photon-to-current efficiency was calculated at 400 nm were calculated for photoanodes with nanopillars of 400 nm in diameter, being 83% for photoanodes onto titanium foils compare to 50% when are deposited on FTO

After stablishing the iron oxide nanopillars φ400 nm, in particular when synthesized on TiO_2_ supports, as the best option among the studied for photoelectrochemical applications, the last step is to check the global photoelectrochemical behavior of this electrode. In this regard, the fabricated electrode was evaluated under solar AM 1.5 G illumination with a power of 55 mW·cm^−2^. The current/photocurrent density versus applied potential curves and the chopped-light chronoamperometry measurements of the electrode are shown in Figures S5 and S6 (supporting information section). Photocurrent density values of the order of 1.4 mA·cm^−2^ were reached at an applied potential of 0.5 V (vs. Ag/AgCl), which imply an increment of 1.6 times compared to the results obtained for the pristine iron oxide nanopillars on FTO substrate under the same illumination conditions. This enhancement of the photocurrent density was because of the mechanism described for the dual effect of Fe_2_O_3_ and TiO_2_ semiconductors. Additionally, TiO_2_ under UV light generates electron-holes contributing to the global photoelectrochemical improvement. The photogenerated holes on TiO_2_ go from the VB of the TiO_2_ to the VB of the Fe_2_O_3_ (see Scheme 1b), and they can easily reach the Fe_2_O_3_/electrolyte interface and generate O_2_ (according to Equation (1)).

These results are even more surprising if one takes into account that light power used was 55 mW·cm^−2^, considering that sunlight power at Earth surface is stablished as 100 mW·cm^−2^ [58]. The state of the art of the iron oxide electrodes on different complex supports [57,59,60] achieved results of the order of 1.5 mA·cm^−2^ by using 100 mW·cm^−2^. Moreover, the amounts of Fe_2_O_3_ present on the electrodes measured by ICP-OES were: 5.03 ± 0.01, 4.95 ± 0.02, 2.45 ± 0.20 and 2.24 ± 0.01 μg per 0.25 cm^2^, for the samples: CL, φ90 nm, φ260 nm and φ400 nm, respectively. Then, the photoelectrochemical results are even more impressive taking into consideration these remarkable small amounts of iron oxide deposited on the electrodes.

## 4. Conclusions

Hematite nanopillars were synthesized by using electron beam evaporation technique with AAO templates. Different controllable diameters, i.e., 90, 260 and 400 nm were obtained according to the AAO templates used. The process is compatible with the use of FTO as support. When using Ti foil, a heterojunction Fe_2_O_3_-TiO_2_ can be established in a single step, while the remaining metallic Ti ensures electrical conductivity and makes possible the use of the nanostructure as electrode. Linear sweep voltammetry shows a clear influence of the photoresponse with the diameters of the Fe_2_O_3_ nanopillars that in general increases with the diameter of the pillars. better photoelectrochemical response utilizing either simulated sunlight and only visible light for the α-Fe_2_O_3_ nanopillars (φ400 nm) with TiO_2_ supports electrodes was obtained, reaching photocurrent density values of the order of 1.4 mA·cm^−2^. The results achieved for the Fe_2_O_3_-TiO_2_ heterojunction represent a clear improvement compared to the prior state of the art on hematite photoelectrodes, increasing the efficiency by at least a factor of two. Thus, the present results show the potential of e-beam nanofabrication in combination with hard macroporous templates to obtain nanostructured hematite electrodes with remarkable solar and visible light photoresponse.

## Data Availability

The data that support the findings of this study are available from the corresponding author upon reasonable request.

## Supplementary Materials

The following are available online at https://www.mdpi.com/article/10.3390/nano11082019/s1, Figure S1: XPS analysis of the iron oxide, Figure S2: XRD analysis of the iron oxide sample after the annealing, Figure S3. Diffuse reflectance UV-Vis spectrum of the hematite sample (a). Tauc’s plots for the calculation of direct (b) and indirect (c) band gap energies, Figure S4. Comparison of photocurrent density under visible light between hematite nanostructures on FTO and Ti foil.
