# Supplementary material for: Optimization of α-Fe2O3 Nanopillars Diameters for Photoelectrochemical Enhancement of α-Fe2O3-TiO2 Heterojunction
Source: Nanomaterials (Basel). 2021 Aug 7;11(8):2019. doi: 10.3390/nano11082019 (PMC8399771; doi:10.3390/nano11082019)
Supplement: Supplementary file 1 [file nanomaterials-11-02019-s001.zip › nanomaterials-1310777-Supplementary Materials.pdf]

Supporting information

Optimization of α-Fe_2O_3 Nanopillars Diameters for Photoelectrochemical Enhancement of α-Fe_2O_3-TiO_2 Heterojunction

Herme G. Baldovi [1,2]

[1] Work address: Department of Chemical Engineering and Biotechnology, University of Cambridge, Philippa Fawcett Drive, CB3 0AS, Cambridge. UK; hergarba@cam.upv.es

[2] Current address: Departamento de Química, Universitat Politècnica de València, Camino de Vera s/n, 46022 Valencia, Spain

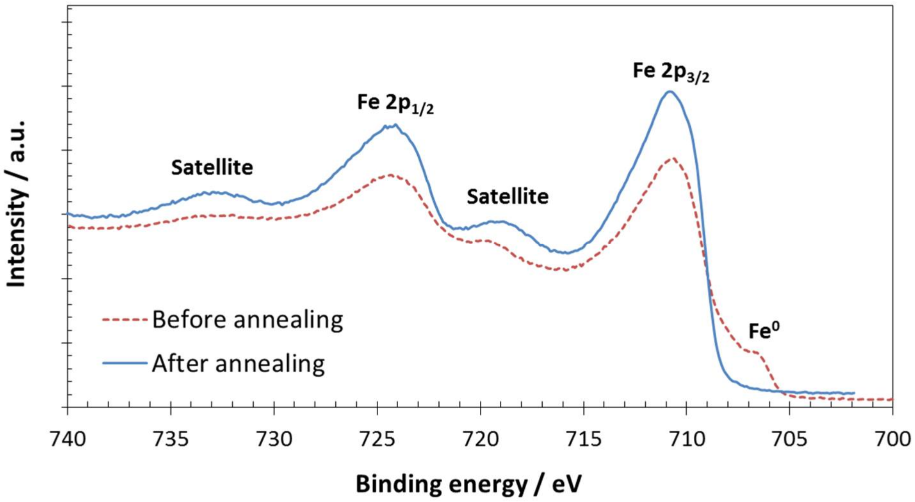

Figure S1. XPS analysis of the iron oxide sample before and after the annealing treatment at 500 °C for 1 with air flow.

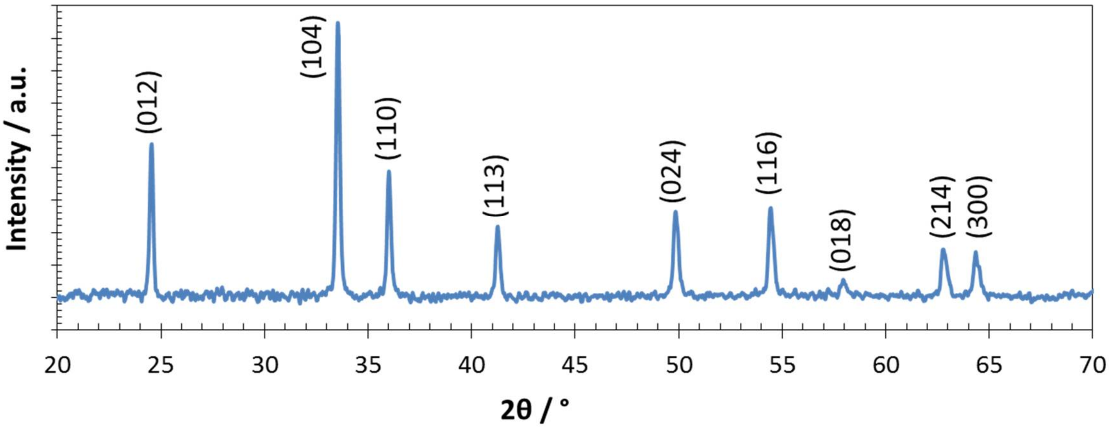

Figure S2. XRD analysis of the iron oxide sample after the annealing treatment at 500 °C for 1 with air flow.

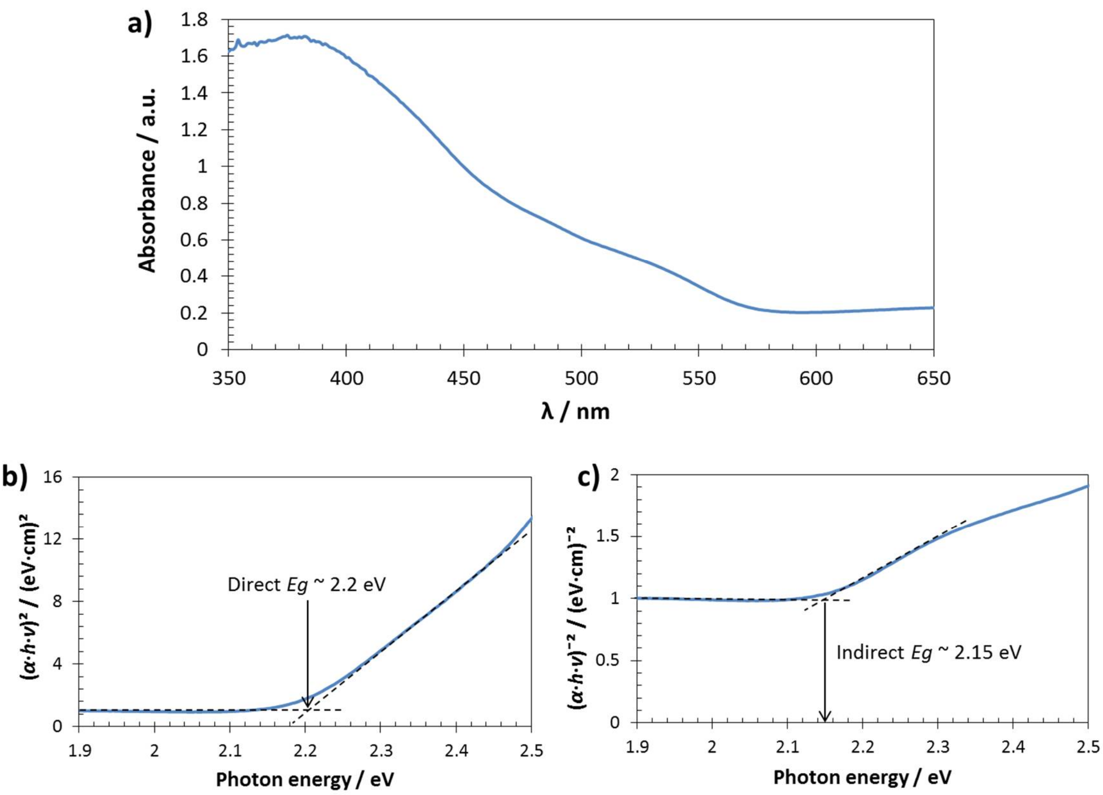

Figure S3. Diffuse reflectance UV-Vis spectrum of the hematite sample (a). Tauc's plots for the calculation of direct (b) and indirect (c) band gap energies.

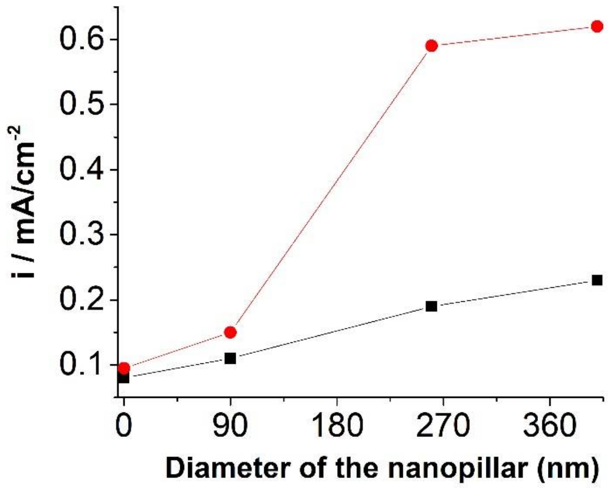

Figure S4. Comparison of photocurrent density under visible light irradiation and applying 0.5 V between hematite samples on FTO (●) and on titanium foil (■) substrates.

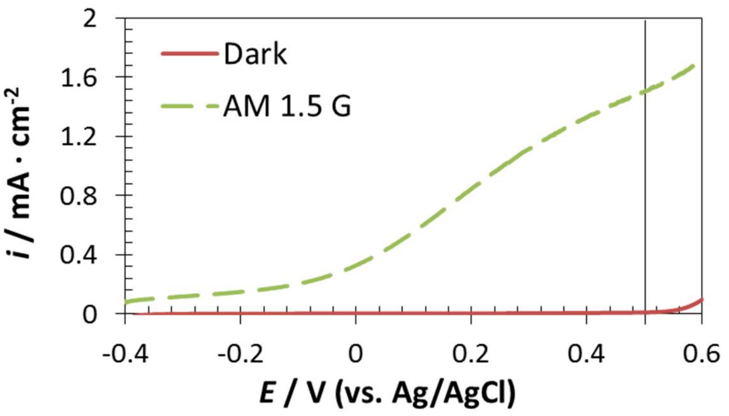

Figure S5. Current/photocurrent density versus applied potential curves of the electrode fabricated on Ti substrates using AAO template φ400 nm. Dark and solar AM 1.5 G (55 mW·cm^{-2}) illumination conditions were applied.

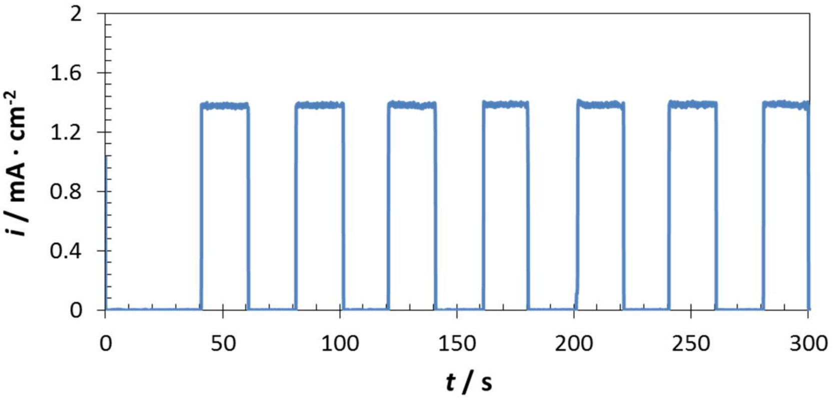

Figure S6. Chopped-light chronoamperometry measurements at 0.5 V (vs. Ag/AgCl) of the electrode fabricated on Ti substrates using AAO template φ400 nm, under solar AM 1.5 G (55 mW·cm^{-2}) illumination.
